# The effect of an expanded long-term periodization exercise training on physical fitness in patients with coronary artery disease: study protocol for a randomized controlled trial

**DOI:** 10.1186/s13063-019-3292-9

**Published:** 2019-04-11

**Authors:** Rita Pinto, Vitor Angarten, Vanessa Santos, Xavier Melo, Helena Santa-Clara

**Affiliations:** 1 Exercise and Health Laboratory, CIPER, Faculdade de Motricidade Humana, Universidade de Lisboa, Estrada da Costa, 1495-687 Cruz Quebrada, Portugal; 2 Ginásio Clube Português, GCP Lab, Praça Ginásio Clube Português 1, 1250-111 Lisboa, Portugal

**Keywords:** Coronary artery disease, Cardiopulmonary exercise testing, Oxygen uptake kinetics, Skeletal muscle oxygenation, Exercise training, Training model

## Abstract

**Background:**

Benefits from cardiac rehabilitation (CR) programs are evidence-based and widely recognized. Less than 50% of people who participate in hospital-based CR programs maintain an exercise regime for as long as six months after completion. Little is known about interventions making the patients continue to exercise after the hospital-based formal program has ended. Methods to ensure sustained benefits of CR need to be tackled. Exercise periodization is a method typically used in sports training, but the impact of periodized exercise to yield optimal beneficial effects in cardiac patients is unclear. Therefore, the purpose of this trial is to evaluate the effects of a long-term exercise periodization on health-related physical fitness components such as cardiorespiratory endurance, muscular strength, skeletal muscle function, and body composition.

**Methods:**

Fifty patients with coronary artery disease will be recruited among those who underwent the hospital-based CR phase. These patients will be randomized (1:1) into one of the following exercise groups: (1) periodized group; and (2) non-periodized group (exercise prescription based on standard guidelines). There will be four assessment time points: at baseline, and 3, 6, and 12 months after starting the exercise training program. At each time point, maximal and submaximal cardiorespiratory fitness, skeletal muscle deoxygenation dynamics, body composition by dual energy radiographic absorptiometry, functional fitness, maximal isometric and dynamic strength, physical activity, and quality of life will be assessed. This experimental design will last for 48 weeks with a frequency of three times per week for both groups.

**Discussion:**

Most medium- to long-term exercise-based CR programs do not employ periodization or exercise progression. Randomized controlled trials are necessary to evaluate long-term periodization outcomes and assess the length of change observed in supervised CR programs. This study will contribute to generate evidence-based exercise prescription approaches to prolong the exercise training after the end of hospital-based CR programs.

**Trial registration:**

ClinicalTrials.gov, NCT03335319. Registered on 22 October 2017.

**Electronic supplementary material:**

The online version of this article (10.1186/s13063-019-3292-9) contains supplementary material, which is available to authorized users.

## Background

Cardiac rehabilitation (CR) is a clinically useful method to modify cardiac risk factors and increase exercise tolerance after cardiac events in patients with coronary artery disease (CAD) [1, 2]. In general, CR programs are performed in three different stages: inpatient CR (phase I); early outpatient CR (phase II); and long-term outpatient CR (phase III) [3]. Most studies on CR have been conducted during phase II and others focused on the follow-up of long-term clinical benefits after the completion of a phase II CR program. Very limited data are available on the effects of CR on health-related physical fitness during a supervised phase III CR.

Exercise adherence after a hospital-based CR program is reported to be poor with only 30–60% of those who complete a phase II CR program still exercising by the sixth month, dropping steadily at the rate of 20–50% by the 12th month [4–7]. The costs of CR are one main reason to this dropout rate.

The existing models of exercise prescription on CR focus on moderate intensity steady state exercises, with walking and cycling being the most recommended types of exercise training (ET) [2]. The repetitive nature of this type of activity can become monotonous for the patient, affecting exercise adherence, compliance, and training outcomes [8]. In this regard, much insight could be gained from periodized approaches used in sport conditioning, designed and planned to be physiologically and psychologically challenging. The classical periodization approach consists of a linear progression, typically moving from general training (high volume/low intensity) towards specific training (low volume/high intensity) [9]. Periodized methods are considered to be superior in strength and power development to non-periodized methods in trained populations [10] and appear to be superior in health outcomes related to traditional and emerging risk factors for cardiovascular disease, low-back and neck/shoulder pain, disease severity, and quality of life in untrained adults [8].

Given the popularity and practicality of periodized training for athletes, it may be feasible and beneficial to prescribe periodized exercise to patients with CAD to improve health outcomes, but this has never been tested. Thus, the purpose of this study is to conduct a 12-month randomized controlled trial (RCT) to evaluate the effects of a linear periodized ET regime versus a non-periodized ET regime (standard exercise prescription guidelines) on peak oxygen uptake (VO_2_ peak), different components of the oxygen kinetics response, oxidative adaptations, maximal isometric and dynamic muscle strength, body composition, functional fitness, and quality of life in patients with CAD.

The primary outcome measure is the change in VO_2_ peak at 12 months. A number of secondary outcome measures will also be assessed, namely the anaerobic threshold (AT), peak work rate, ventilatory equivalent of carbon dioxide (VE/VCO2), ventilatory equivalent of oxygen (VE/VO2), minute ventilation, oxygen kinetics, muscle deoxygenation dynamics of the vastus lateralis muscle at the submaximal test, maximal strength, handgrip strength, total body bone mineral content, lean and fat tissue mass, percentage of body fat mass, functional fitness score, moderate and vigorous physical activity, and quality of life.

Considering that this type of linear periodization exerts higher stress on the cardiovascular and neuromuscular systems, we hypothesize that higher increases in VO_2_peak, muscle strength, body composition, and functionality will be found in the periodized ET regime compared to non-periodized ET regime. In addition, we hypothesize that improvements in microvascular O_2_ delivery in the exercise transient in response to periodized ET regime will be superior to those of the non-periodized ET regime.

## Methods/Design

### Study design

**Fig. 1:**
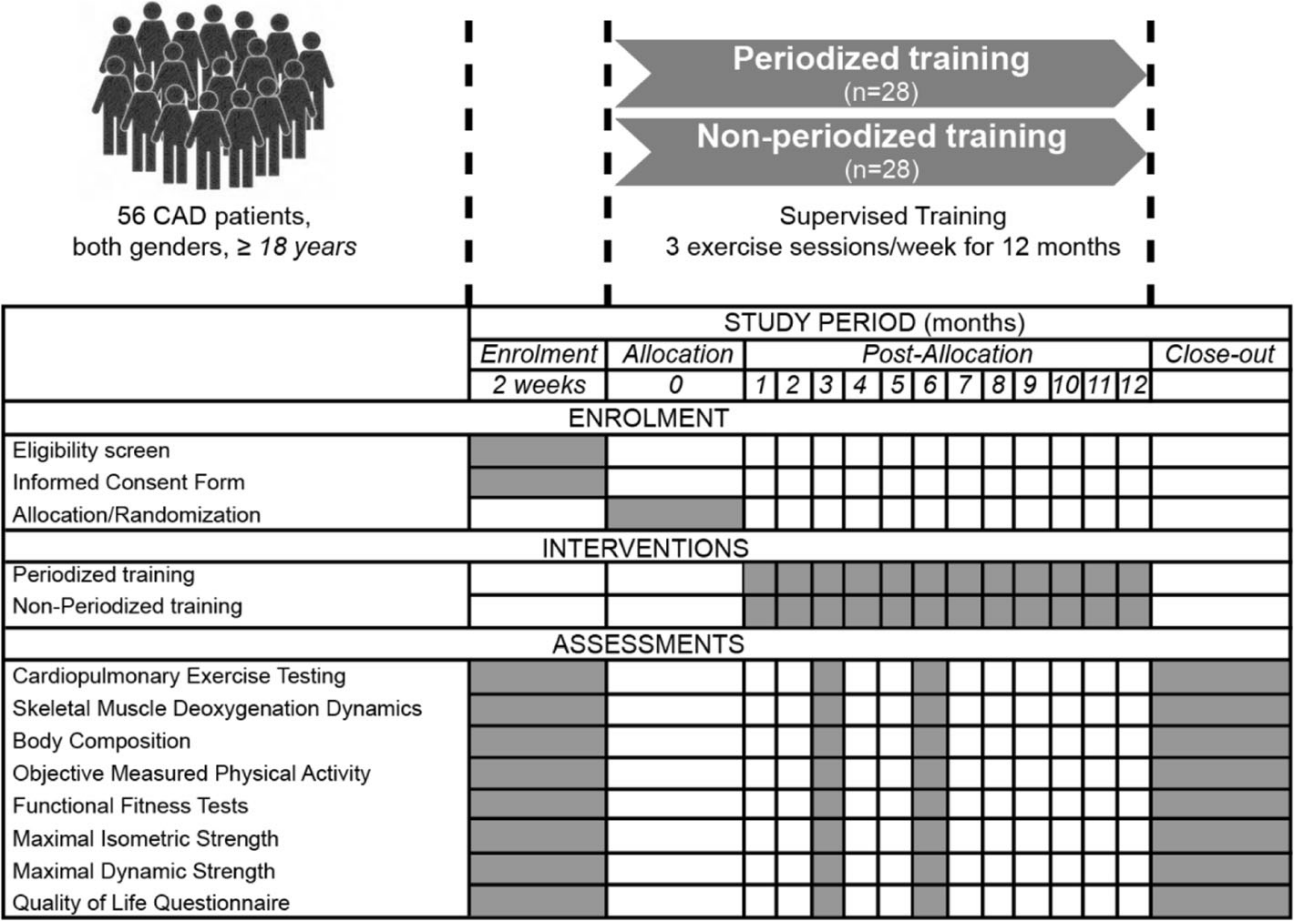
Standard Protocol Items: Recommendations for Interventional Trials (SPIRIT) figure showing the schedule of enrolment, intervention, and assessments

This is a longitudinal randomized study with two distinct ET prescriptions (linear periodization vs non-periodization) specifically designed for patients with CAD (Fig. 1). Participants will exercise over a period of 12 months, with a frequency of three times per week. Briefly, following consent, patients will be randomized 1:1 and stratified (by sex, age, and VO_2_peak at baseline) into either one of two groups: linear periodization or non-periodization. Each stratification factor has two levels: (1) sex: male and female; (2) age: < 65 years and ≥ 65 years, considering the term older adult defines individuals aged ≥ 65 years [11]; VO_2_ peak: above or below the predicted VO_2_ peak for patients with combined medical and surgical diagnoses entering in a CR program [12]. The regression equation for predicted VO_2_ peak for men is as follows: 33.970 – 0.242 × age. For women, the equation is: 21.693 – 0.116 × age. A randomization code will be developed with a computer random-number generator to select random permuted blocks. Approximately equal numbers of women and men will be allocated to the linear periodization or non-periodization groups. To ensure allocation concealment, researchers will only request randomization after completion of all baseline assessments. Differential treatment or assessment of participants potentially resulting in bias may occur at any phase of the present trial [13].

At least three out of an ideal five groups of individuals involved in this trial will be blinded: participants; exercise physiologists (prescribing and supervising the exercise sessions); data collectors; outcome adjudicators; and data analysts. Because both interventions are structured in nature, it is not possible to perform patient blinding. Exercise physiologists, who prescribe and supervise exercise sessions, also cannot be blinded; however, they will not be involved in data analysis or reporting. All assessments will be performed in a blinded format by operators unaware of randomization. Since bias may also be introduced during the statistical analysis of the trial through the selective use and reporting of statistical tests, data analysts will be blinded until the entire analysis is completed [13]. Measurements of the primary and secondary outcomes will take place at M0–baseline, M1–3 months after starting the ET, M2–6 months after starting the ET, and M3–12 months after starting the ET. Medication or changes in prescribed therapeutic will be registered at each time point. We will not give any particular advice regarding diet or other lifestyle factors during the trial. However, we will ask all participants to maintain their normal diet during the intervention period. In order to assure the confidentiality of the participants, an ID code will be attributed to each participant in the database and all the equipment and spreadsheets used. A single researcher will perform the database management.

### Participants

Patients with CAD who underwent a hospital-based CR program in any hospital within the Lisbon district will be recruited. The study will be performed at the Gym of the University Stadium of Lisbon, clinically supervised by the Faculty of Medicine from the University of Lisbon. Inclusion criteria include: age > 18 years; male and female participants who had an established history of the following conditions or procedures: angiographically documented CAD in at least one major epicardial vessel; those that had clinical evidence of CAD in the form of previous MI; or coronary revascularization (coronary artery bypass grafting [CABG] or percutaneous coronary intervention); or angina pectoris. Exclusion criteria will be participants who have heart failure, had heart transplants with either cardiac resynchronization therapy or implantable defibrillators, and inability to comply with guidelines for participation in exercise testing and training [14] as well as significant limiting and/or unstable co-morbidities that would prevent full participation such as other diagnosed cardiovascular disease, arthritis, and/or metabolic disorders. Patients will be excluded from the study if a new cardiac event develops (myocardial infarction, coronary stent insertion, coronary angioplasty, or CABG surgery), hospitalization or any physical limitation that would prevent from exercising. Relative and absolute contraindications for exercise testing will be assessed before each measurement round followed by strict compliance with the indications for termination of exercise testing [15]. Compliance with the ET and adherence will be determined by the number of training sessions attended and successfully completed in accordance with the exercise protocol. It is common among CR studies to establish a minimum training attendance of at least 75%, set upon the minimum attendance rate to attain expected results [16]. Participants who attend at least 75% of their scheduled exercise sessions will be assigned as adherent and participants who attend < 75% of the exercise sessions as non-adherent.

To verify the safety on both training groups, adverse and serious adverse events will be carefully monitored, recorded, and reported in line with the principles of Good Clinical Practice [17]. Immediate on-site reports will be followed by detailed written reports. The immediate and follow-up reports will identify individuals by unique code numbers assigned to the trial participants rather than by their names, personal identification numbers, and/or addresses. In line with the principles of Good Clinical Practice, the nature and severity of the event, in addition to its potential association with the ET intervention, will be ascertained by the principal investigator and ratified by the clinical director of the CR program [17]. Standard clinical examination will include medical history and cardiovascular risk factor assessment, that is, resting blood pressure, diabetes, family history of premature cardiovascular heart disease, and smoking status.

### Sample size

Sample size was calculated (G-Power, *Version 3.1.3)* assuming a difference in VO_2_ peak between groups of 3.5 mL/kg/min to be clinically relevant, where each 1 MET (3.5 mL/kg/min) increase in cardiorespiratory fitness is associated with an 8–35% reduction in overall mortality at 12 months [18–20]. Assuming a VO_2_ peak standard deviation of 3.5 mL/kg/min, α = 0.05, 1-β = 0.80, and an expected dropout rate at 12 months of 37% [4], the calculations yielded a total minimum sample size of 50 participants (25 in each group).

### Intervention

#### Exercise training program

The ET program will be carried out mostly in groups at the Gym of the University Stadium of Lisbon, three times a week (60 min per session) on non-consecutive days for 48 weeks (average of 120 exercise sessions) and supervised by exercise physiologists for both groups. To ensure similar training loads (i.e. volume × intensity) between groups despite differences in intensity, training impulses (TRIMP) will be used according to the method by Edwards [21] for the aerobic component, and according to the volume load method for the resistance training component [22, 23]. All sessions will include 10 min of warm-up and cool-down exercises standardized for both groups. The warm-up will focus on pulse raising, mobility, and preparatory stretching for the conditioning component. Cool-down will include transition from conditioning component to the stretching phase, which comprises static and dynamic stretching exercises for all major muscle groups. Sessions will be deemed completed when at least 90% of the prescribed exercises have been successfully performed.

##### Linear periodized group

**Fig. 2:**
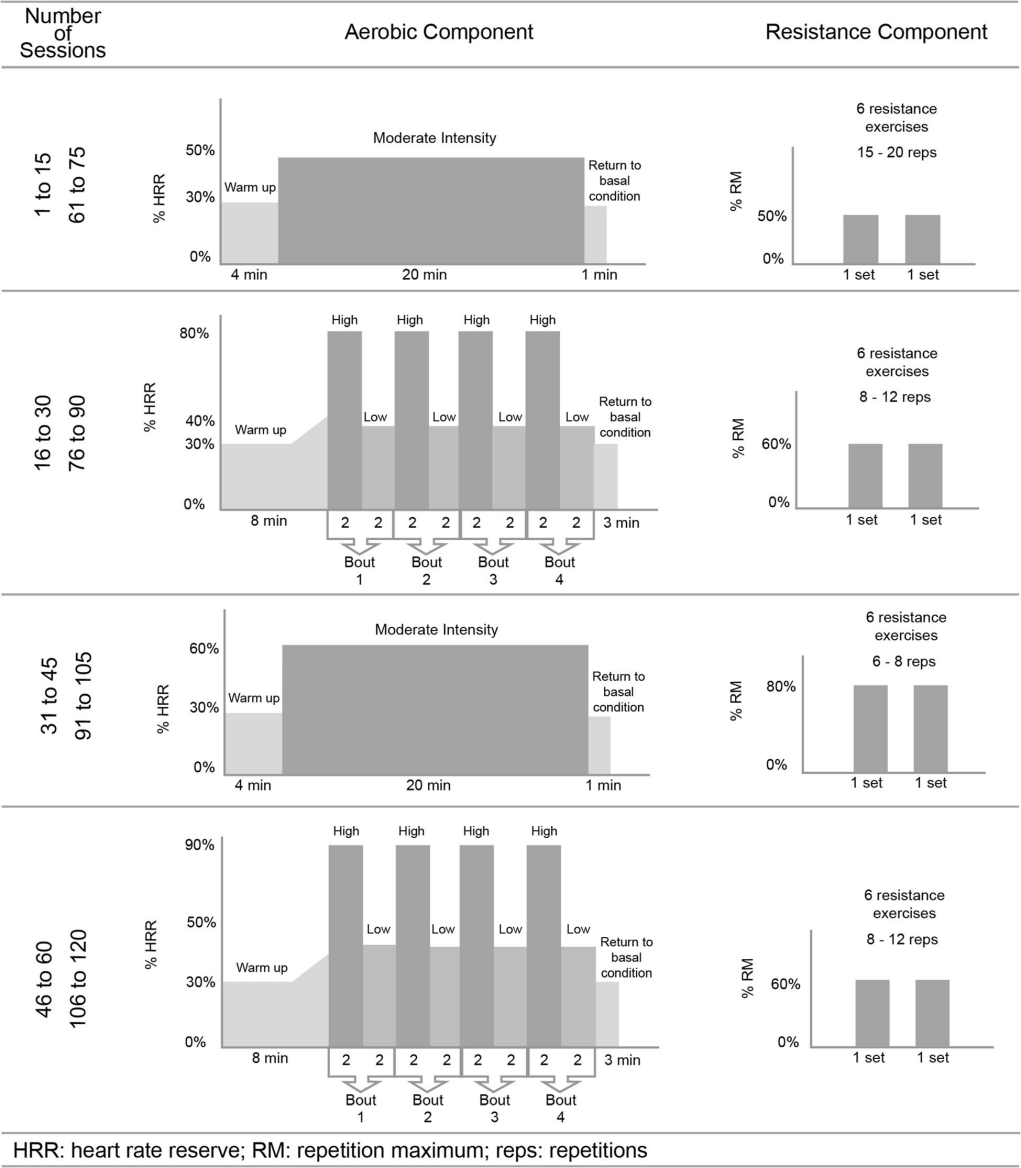
Periodized exercise prescription on the aerobic and resistance component for 12 months

The exercise prescription will be gradually progressed through various combinations of duration, frequency, and/or intensity of training (Fig. 2). Over the first 15 exercise sessions, 20 min of continuous exercise on an ergometer (cycle ergometer or treadmill) will be prescribed with the intensity set on the first AT (AT1) determined from the cardiopulmonary exercise test (CPET) or 50–60% of the heart rate reserve (HRR), if the AT could not be adequately determined [24], Borg Rating of Perceived Exertion (RPE) equivalent 11–12; followed by two sets of 15–20 repetitions of resistance training on major muscle groups at 50% of 1 repetition maximum (RM); from the 16th to the 30th session, consisting of high-intensity interval training (HIIT) [25]: four interval training periods of 2 min (second AT [AT2] intensity or 80–90% HRR, if AT2 could not be determined [24], RPE > 14) and four active pauses of 2 min (below AT1 or 40–50% HRR, RPE 10–11) between interval training periods, followed by two sets 8–12 repetitions of resistance training on major muscle groups at 60% 1RM. From the 31st to the 45th exercise session, 20 min of moderate continuous training (MCT) will be prescribed at an intensity of 60–70% HRR, RPE 12–13, followed by two sets of 6–8 repetitions of resistance training on major muscle groups at 80% 1RM. From the 46th to the 60th exercise sessions, HIIT will be prescribed once again but at an intensity above AT2 or > 90% HRR, RPE > 16 and the active pauses will be at AT1 or 50–60% HRR, if AT1 could not be determined, RPE 11–12, followed by two sets of 8–12 repetitions of resistance training on major muscle groups at 60% 1RM. At the end of the 60th session, an exact copy of the past mesocycle will be prescribed until the 120th session. The three types of resistance training prescribed will focus on the major trunk and upper and lower body muscle groups on the following machines (Life Fitness, Rosemont, IL, USA): leg curl; leg press; leg extension; chest press; lateral pull down; and low row.

##### Non-periodized group

**Fig. 3:**
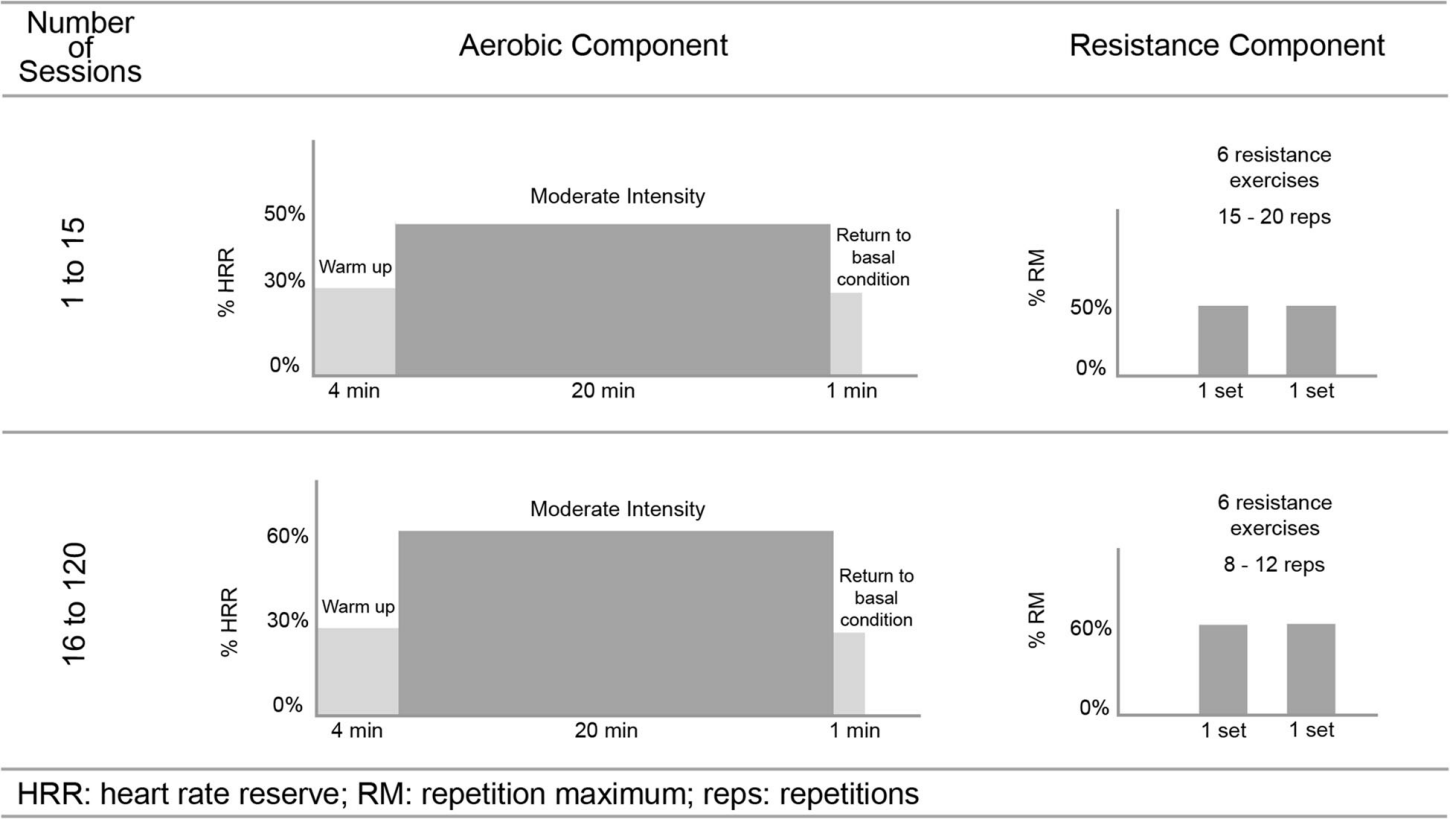
Non-periodized exercise prescription on the aerobic and resistance component for 12 months

A combined ET regime (aerobic and resistance training) will be prescribed according to the ACSM Guidelines 2017 [14]. MCT will be prescribed at an intensity of 40–75% HRR to be performed three days each week on non-consecutive days, for 20 min per session, using available ergometers (cycle ergometer or treadmill). Resistance training will be performed after the aerobic component to allow for an adequate warm-up. Patients will start with 10–15 repetitions at ~ 30–40% 1RM for the upper body and ~ 50–60% 1 RM for the lower body. Low-risk patients may progress to 2–4 sets of 8–12 repetitions with resistances of ~ 60–80% 1RM with a rest interval of 2–3 min between sets. Each major muscle group (i.e. chest, shoulders, arms, abdomen, back, hips, and legs) should be trained initially with one set; multiple set regimens may be introduced later as tolerated (Fig. 3).

By design, the non-periodized group involves an identical total training volume and time commitment but distinct metabolic stress compared to the linear periodized group. All patients will be monitored with a heart rate (HR) monitor (H7 Polar, Electro, Kempele, Finland) during the exercise sessions. The resistance machines will be the same as mentioned previously for both groups. Blood pressure will be assessed before and after completing each session. If necessary, the blood pressure will be measured during the ET session. All the patients will be instructed on the correct exercise techniques and to avoid the Valsalva maneuver.

### TRIMP calculations

The quantification of the training load in the aerobic component will be performed according to the methods by Edwards [21] dividing the efforts in several HR intensity zones (HRmax) - Zone 1: 50–60% of HRmax; Zone 2: 60–70% of HRmax; Zone 3: 70–80% of HRmax; Zone 4: 80–90% of HRmax; and Zone 5: 90–100% of HRmax. Since the proposed HR prescription is based on % HRR, % HRmax will be converted to % HRR (Table 1).

**Table 1:** Methods of estimating intensity of cardiorespiratory exercise

Intensity	%HRR or %VO_2_R	%HR max	Edwards method Zone
Very light	< 30	< 57	1
Light	30–< 40	57–< 64	2
Moderate	40–< 60	64–< 76	3
Vigorous	60–< 90	76–< 96	4
Near maximal to maximal	≥ 90	≥ 96	5

*HRR* heart rate reserve, *VO*_*2*_*R* oxygen uptake reserve, *HRmax* maximal heart rate

Adapted from Garber et al. [38]

In the TRIMP calculation, the time accumulated in each HR zone is multiplied by its value and the results obtained will be summed (Table 2). The equation for calculating the volume load for the resistance training is accomplished by multiplying the number of repetitions by the percentage of 1 RM [23]. This equation is represented as follows: *Volume load (kg) = number of sets × number of repetitions × %1RM.* In both groups, TRIMP is similar (linear periodized: 11,320 vs non-periodized: 11,350).

**Table 2:** Intensity and duration, as quantified by daily TRIMP per every 15 exercise sessions during the training period for the linear periodized group and the non-periodized group

Sessions (n)	Total time	Aerobic component												
		Linear periodized group									Non-periodized group			
		Work sets	Work (min)	Rest sets	Rest (min)	Work (zone)	Rest (zone)	Extra (min)	Extra (zone)	TRIMP daily	Work sets	Work (min)	Work (zone)	TRIMP daily
1–15	20	1	20	0	0	2	0	0	0	40	1	20	2	40
16–30	20	4	2	4	2	4	2	4	1	52	1	20	3	60
31–45	20	1	20	0	0	3	0	0	0	60	1	20	3	60
46–60	20	4	2	4	2	5	3	4	1	68	1	20	3	60
61–75	20	1	20	0	0	3	0	0	0	60	1	20	3	60
76–90	20	4	2	4	2	4	2	4	1	52	1	20	3	60
91–105	20	1	20	0	0	3	0	0	0	60	1	20	3	60
106–120	20	4	2	4	2	5	3	4	1	68	1	20	3	60
										460				460

### Measurements

#### Cardiopulmonary exercise testing

CPET is the gold-standard technique to measure maximal and submaximal functional capacity. These tests will be performed with the participants in a non-fasting condition and under regular medication.

##### Symptom-limited exercise test

A symptom-limited ramp incremental CPET will be performed according to the Clinician’s Guide to CPET in Adults [26], in a cycle ergometer (CardioWise Ergo Fit, Pirmasens, Germany) using a breath-by-breath gas analyzer (Ergostik, Geratherm Respiratory GmbH, Bad Kissingen, Germany). Resistance will increase 10–25 W/min every minute on a constant pedal frequency of 60 rotations/min. Each patient will be encouraged to exercise to exhaustion, as defined by intolerance, leg fatigue, or dyspnea, unless clinical criteria for test termination occurred. Patients will remain seated on the cycle ergometer as soon as they stop, while recovery measurements are taken. Twelve-lead ECG will be recorded continuously (Mortara X-Scribe eletrocardiograma instrument Inc., Milwaukee, WI, USA) and blood pressure will be recorded at baseline, every 2 min, at peak exercise, and during recovery. Peak oxygen capacity will be considered the highest attained VO_2_ during the final 30 s of exercise and AT will be estimated by the V-slope method. HR recovery as a simple marker of parasympathetic activity will be calculated as the difference between peak HR and HR 1 min later. All patients should achieve a respiratory exchange ratio of > 1.1, an indicator of maximal effort in the CPET.

##### Submaximal exercise test

On a separate day, patients will be recruited for a constant load exercise test on the same cycle ergometer and gas analyzer. The test load will be set at 80% of the work rate corresponding to the AT determined from the symptom-limited ramp incremental test or at 50% of VO_2_ peak if AT could not be adequately determined. Cardiopulmonary data will be recorded for 2 min at rest followed by 1 min of unloaded pedaling. After unloaded pedaling, patients will perform the constant load test for 6 min at the work rate described above, followed by a passive recovery of 5 min. To improve the confidence of the kinetic parameter determination, this test will be performed three times and averaged.

Curve fitting will be performed with Graphics software Origin version 7.0 (Microcal Software Inc., Northampton, MA, USA) using interactive techniques. The kinetics of the oxygen uptake response to the constant workload exercise will be analyzed by the sequent separate phases.

Before each maximal and submaximal test, the gas analyzer will be calibrated using ambient air and standard calibration gases of known concentration (16.7% O_2_ and 5.7% CO_2_). The calibration of the turbine flowmeter will be performed using a 3 l syringe (Quinton Instruments, Seattle, WA, USA).

### Skeletal muscle deoxygenation dynamics

The muscle deoxygenation dynamics of the Vastus Lateralis muscle will be evaluated throughout the submaximal CPET. Deoxyhemoglobin (HHb), oxyhemoglobin (HbO_2_), and total hemoglobin (HbT) concentrations will be quantified with a continuous-wave tissue oximeter (NIMO, Nirox srl, Brescia, Italy), based on the near-infrared spectroscopy (NIRS) system, which provides continuous, non-invasive monitoring of the relative concentration changes in these variables during rest and exercise. Briefly, this system is based on the oxygen dependency of absorption changes for NIR light in hemoglobin and myoglobin; it consists on an emission probe which emits three wave lengths (685, 850, and 905 nm) and a photon detector. The intensity of incident and transmitted light will be record continuously at 40 Hz and used to estimate the concentrations changes relative to baseline for oxygenated, deoxygenated, and total hemoglobin. To account for the possible influence of the local fat layer on NIRS a real-time correction using an algorithm included in the software program v2.0 supplied with the spectrometer (Nimo Data Analysis Peak) will be used. Since the HHb signal is less dependent of changes in blood flow it can be used as an indicator of fractional O_2_ extraction within the microvascular level.

This task will be performed at rest, during, and immediately after the submaximal CPET. The off-oxygen consumption and HHb kinetics will be determined using a monoexponential model which incorporates an amplitude, time constant, and time delay.

### Body composition – dual-energy radiographic absorptiometry (DXA)

All patients will be tested in the morning following a 12-h fast and refrained from caffeine, alcohol, and moderate to vigorous exercise during the last 24-h. Total and regional body mass (bone mineral content, lean soft tissue, and fat mass) will be estimated using dual energy radiographic absorptiometry (DXA) (Hologic Explorer-W, fan-beam densitometer, software QDR for windows version 12.4, Hologic, USA). The attenuation of X-rays pulsed in the range of 70–140 KV synchronously with the line frequency for each pixel of the scanned image. The same lab technician will perform the scans and execute the analyses according to the standard analysis protocol. Height will be measured to the nearest 0.5 cm with a stadiometer (SECA, Hamburg, Germany), body weight will be measured on a weight scale (SECA, Hamburg, Germany), and the body mass index (BMI) will be calculated (kg/m^2^). Waist circumference will be measured using an inelastic flexible metallic tape (Lufkin W606 PM, Vancouver, Canada) to the nearest 0.1 cm. All anthropometric procedures will be led by the same certified technician.

### Objective measured physical activity

Each participant will be asked to wear the accelerometer ActiGraph GT3X+ (AG; ActiGraph, Pensacola, FL, USA) for the following seven days. The ActiGraph GT3X+ will be attached to an elastic waist belt and placed in line with the axillary line of the right iliac crest. Participants will be asked to wear the accelerometer from the moment they wake up until they go to bed at night and requested to remove it only during water-based activities such as showering and swimming and during sleep. ActiGraph GT3X+ will be initialized using a sample rate of 30 Hz and then downloaded using the low filter extension option in Actilife5 Software v5.7.4 (ActiGraph, Pensacola, FL, USA). The cut-off points previously used in an older sample of adults to calculate daily times in each activity intensity band will be: sedentary (< 1.5 MET) 0–199 counts per minute (cpm); light (1.5–3 MET) 200–1998 cpm; and moderate to vigorous physical activity (> 3 MET): ≥1999 cpm. Sensitivity analyses will also be performed using a more conservative cut point of 0 cpm to differentiate sedentary time from activity. All physical activity variables will be converted to time (in minutes) per valid day.

### Functional fitness tests

The functional fitness tests are a simple, reproducible, readily available tool frequently employed to assess submaximal functional capacity and evaluate the response to intervention [27]. The 6-min walking test will be performed indoors, along a long flat, straight, enclosed 20-m corridor with a hard surface that is seldom. Patients will be instructed to walk at their own pace according to their tolerance to exercise for 6 min, with rest stops as needed. The final result will be the distance in meters covered during the 6-min test. Total distance during the test will be recorded. The 30-s chair stand assesses the lower body strength, needed for numerous tasks such as climbing stairs or walking. Patients will be instructed to sit and stand as fast as they can in 30 s with arms folded across the chest. The 8-ft (2.44 m) up and go test evaluates agility/dynamic balance, which is important in tasks that require quick maneuvering. It will be evaluated the time in seconds that the participant needed to get up off the chair, walk the distance of 2.44 m, and return to the initial position (seated). Upper and lower body flexibility will be measured with the back scratch test and the chair sit-and-reach, respectively. During the back scratch test, the number of centimetres between middle fingers (+ and −) will be measured while reaching over the shoulder with arm and up the middle of the back with the other arm. For the lower body flexibility, from a sitting position at the end of a chair, with one leg extended and hands reaching toward toes, the number of centimetres (+ or −) between extended fingers and tip of toe will be measured.

### Maximal isometric strength

Maximal handgrip strength will be assessed by a portable hand dynamometer JAMAR plus digital (Sammons Preston, Bolingbrook, IL, USA). The patients will be all positioned according to the American Society of Hand Therapists guidance [28]. Briefly, the handgrip test will be performed with the patients in a seated comfortable position, with the shoulder adducted and close to, but not supported by, the trunk. The elbow of the assessed limb should be flexed to 90° and the forearm should be in a neutral position (halfway between supine and pronation position). A variation of 0–30° in the wrist extension will be allowed. Each participant will attempt three maximal measures on both hands alternately. After each attempt, there will be a resting period of 60 s that will be used both for recovery and for changing the handgrip dynamometer to the opposite hand. All patients will be instructed not to perform a Valsalva maneuver during the tests.

### Maximal dynamic strength

Maximal strength will be assessed by 1RM test for each of six weight exercises on variable resistance machines (Life Fitness, Rosemont, IL, USA) available at the Gym of the University Stadium of Lisbon as follows: leg press; leg extension; leg curl; low row; chest press; and lateral pull down. The protocol test of 1RM will be determined as previously described in our prior studies [29]. Strength will be recorded as the maximal number of kilograms lifted in one full range of motion. The order of the tests will be the same for all patients.

### Quality of life questionnaire

The Short Form-36 Health Survey (SF-36) is a self-assessment health status questionnaire composed of 36 questions about sociodemographic, health, and personal behavior [30]. It was designed for use in clinical practice and research, health policy evaluations, and general population surveys. The 36 questions in the SF-36 survey capture the individual’s perception of their general health by sorting them into multi-item scales that assess eight concepts. The eight subscales are as follows: physical functioning (10 questions); role/physical (4 questions); bodily pain (2 questions); general health (10 questions); vitality/energy (4 questions); social functioning (2 questions); role/emotional (3 questions); mental health/emotional wellbeing (5 questions). The SF-36 also provides two important summery measures of health-related quality of life: physical component summary and mental component summary scales. The strength of both scales lies in their ability to distinguish a physical from a mental outcome. The items and dimensions in SF-36 were constructed using the Likert method of summated ratings. This questionnaire has been used in CR programs [31]. A Portuguese validated version of SF-36 is available [32].

### Data analysis

Primary and secondary outcome variables will be presented as means ± standard deviation. Missing data points will be assessed for random occurrence using Little’s Missing Completely at Random (MCAR) and then imputed using expectation-maximization algorithms. Normality and homogeneity will be tested using Shapiro–Wilk test and Levene’s test, respectively. Generalized estimating equations will be used to estimate the between-group and within-group effects on primary and secondary outcomes, while allowing control for potential confounders (i.e. medication, exercise training compliance, physical activity).

Statistical significance will be set at an alpha level of 0.05. Statistical analyses will be conducted using Statistical Package for the Social Sciences (SPSS) 24.0 (IBM SPSS Statistics, Chicago, IL, USA).

### Ethical and legal issues

Informed consent will be obtained before entry in the trial. The study was approved by the Faculty of Human Kinetics, University of Lisbon Ethics Council (approval number: 30/2017) and conducted in accordance with the Declaration of Helsinki [33]. This trial has been registered at ClinicalTrials.gov (NCT03335319).

## Discussion

ET is recognized as a valuable adjunct therapeutic in the management of several chronic cardiac conditions, with well-established benefits such as improvements in exercise capacity and quality of life [2]. However, maintaining or improving those benefits on a long-term CR program remain unknown and an ongoing challenge. The majority of exercise intervention studies in patients with CAD examined the effects of short-term (up to three months) CR programs. Follow-up studies consistently report poor adherence to physical activity recommendations [34, 35], high drop-out rates, and a decline in exercise capacity in the long term [36]. Considering that a great part of favorable hemodynamic, cardiorespiratory, and musculoskeletal adaptations are lost within three months of exercise cessation [37], it is essential for patients with CAD to follow a regular and uninterrupted exercise program throughout life.

To our knowledge, there are currently no studies where this particular design has been used to test whether the combination of cardiovascular and strength periodization ET can elicit better effects on exercise capacity, strength, physical function, body composition, physical activity, and quality of life compared to current guidelines (no periodization). Multiple training variables can be manipulated during exercise prescription, including repetitions, interval length, rest period length, and intensity of resistance. We believe much insight could be gained from approaches used in sport conditioning, where exercise prescription is designed to be physiologically and psychologically sustainable using periodization. If it shows to be true, the results of this study might have important implications for future ET prescription optimization in outpatient CR programs. This study is not without limitations. Although we tried to reduce bias by turning the evaluators and data analysts, blinded to the randomization. We will also control the results for potential confounders such as medication, physical activity, and ET compliance. Medication or changes in prescribed therapeutic will be registered at every time point. We will not give any particular advice regarding diet or other lifestyle factors during the trial. However, we will ask all participants to maintain their normal diet during the study duration. Objective measured physical activity will be assessed at baseline, and at 3, 6, and 12 months. As mentioned before, all exercise sessions will be deemed completed when at least 75% of the prescribed exercises have been successfully performed. All completed sessions will be counted and registered one by one. The long-term effect of community-based CR programs will remain challenging due to scarce availability of such programs and the inadequate referral by healthcare practitioners. The time-consuming events related to the assessment of different variables will require extra time and planning, in addition to other activities related to the exercise program, requiring the attendance of the participants.

The major strength, to our knowledge, will be that this is the first RCT to analyze the long-term effects of a supervised periodized ET prescription in patients with CAD, which provide knowledge in better understanding what can be another option on ET prescription. The intervention given in the study will be designed as a “realistic intervention. We have designed an intervention to address reported barriers to exercise-based CR programs [33]. Our CR program has unique features that allow us to anticipate high levels of adherence: (1) the clinical director of outpatient CR program is also the clinical director of the hospital-based CR program at the Centro Hospitalar Universitario Lisboa Norte; (2) all assessments are free of charge to all participants in this trial; (3) our program runs on flexible schedules (from 8 am to 8 pm); (4) it is a supervised exercise-based CR program; (5) the gym is at a short distance walk from public transportation (metro and bus) with a private parking lot near the gym; and 6) is in the vicinity of Santa Maria University Hospital.

Nonetheless, hopefully the results of the present study will be important to suggest that ET programs should be supervised and personalized as much as possible to optimize health outcomes, reduce dropouts, and to increase adherence.

## Trial status

Recruitment and screening will last from September 2017 to December 2018 and assessments will continue to December 2019.

## Additional file

Additional file 1: SPIRIT 2013 Checklist: Recommended items to address in a clinical trial protocol and related documents. (DOC 124 kb)

## Abbreviations

AT: Anaerobic threshold
CAD: Coronary artery disease
CPET: Cardiopulmonary exercise training
CR: Cardiac rehabilitation
DXA: Dual-energy radiographic absorptiometry
ET: Exercise training
HIIT: High-intensity interval training
HR: Heart rate
HRR: Heart rate reserve
MCT: Moderate continuous training
NIRS: Near-infrared spectroscopy
1 RM: One repetition maximum
RPE: Borg rating of perceived exertion
TRIMP: Training impulses
VO2 peak: Peak oxygen uptake
